# Asprosin activates multiple placental pathways *in vitro*: Evidence for potential involvement in angiogenesis, fatty acid metabolism and the mTOR, NOTCH and WNT signalling pathways

**DOI:** 10.3892/mmr.2025.13674

**Published:** 2025-09-04

**Authors:** Sophie Orton, Seley Gharanei, Jovile Kazileviciute, Sayeh Saravi, Vanlata Patel, Jayanta Chatterjee, Ioannis Kyrou, Emmanouil Karteris, Harpal S. Randeva

**Affiliations:** 1 Warwick Medical School, University of Warwick, Coventry CV4 7AL, UK; 2 College of Health and Life Sciences, Aston Medical School, Aston University, Birmingham B4 7ET, UK; 3 College of Health, Medicine and Life Sciences, Brunel University of London, Uxbridge UB8 3PH, UK; 4 Academic Department of Gynaecological Oncology, Royal Surrey NHS Foundation Trust Hospital, Guildford GU2 7XX, UK; 5 Warwickshire Institute for The Study of Diabetes, Endocrinology and Metabolism, University Hospitals Coventry and Warwickshire NHS Trust, Coventry CV2 2DX, UK; 6 Centre for Sport, Exercise and Life Sciences, Research Institute for Health and Wellbeing, Coventry University, Coventry CV1 5FB, UK; 7 College of Health, Psychology and Social Care, University of Derby, Derby DE22 1GB, UK; 8 Laboratory of Dietetics and Quality of Life, School of Food and Nutritional Sciences, Agricultural University of Athens, 11855 Athens, Greece

**Keywords:** asprosin, gestational diabetes mellitus, TLR4, placenta, protein tyrosine phosphatase receptor type D

## Abstract

Asprosin is glucogenic adipokine that exerts a wide repertoire of actions, including the regulation of appetite, insulin resistance and cell proliferation. At present, little is known about the actions of asprosin in the human placenta. The present study investigated the effects of asprosin on the transcriptome of the BeWo and JEG-3 p2lacental cell lines, and assessed the expression of FBN1/Furin and asprosin's candidate receptors in healthy placentas when compared against placentas from pregnancies where the carrier had gestational diabetes mellitus (GDM). A number of methods, including tissue culture, clinical sample collection, RNA extraction, RNA sequencing, reverse transcription-quantitative PCR and gene enrichment analyses were used in the present study. RNA sequencing revealed that asprosin induced cell specific differential expression for 51 genes in BeWo cells, and 204 in JEG-3 cells, with nine common differentially expressed genes in both *in vitro* models including *SLCA1* and *HK2*. Specific pathways involved in angiogenesis, fatty acid metabolism and mTOR/NOTCH/WNT/p53 signalling were also enriched. Only *TLR4* was significantly downregulated in GDM placentas when compared with controls. The present study provides novel insight into the actions of asprosin in two well-established *in vitro* placental (trophoblast) models, identifying key genes and signalling pathways. A common theme identified from these findings is that of glucose homeostasis, in accordance with the role of this adipokine.

## Introduction

The human placenta is a transient foetal organ during pregnancy which is responsible for multiple vital functions, including the transfer of nutrients/factors/gases between the mother and the foetus, the removal of waste products from the foetus, and immunoprotection for the foetus (1,2). Among these functions, the placenta also acts as an endocrine organ, secreting a number of key hormones (e.g., steroids) for the maintenance of pregnancy and foetal development (1,2).

Asprosin was identified by Romere *et al* (3) during investigation of Neonatal Progeroid Syndrome (NPS), showing that its pathogenesis is due to premature ablation of profibrillin-1 (pro-FBN1) by furin (3). Patients with NPS have a unique phenotype characterised by extreme leanness, low appetite, lipodystrophy and insulin sensitivity. Asprosin is implicated in a number of physiologic processes and is perhaps most known for its glucogenic function, namely stimulating hepatic glucose secretion via the G-protein-cAMP-PKA pathway (4). The receptor by which asprosin carries out its glucogenic function has been established to be the mouse olfactory receptor OLFR743, whilst in humans asprosin is thought to act via the ortholog OR4M1 (4,5). Asprosin has also been found to induce pro-inflammatory effects in both skeletal muscle and pancreatic β cells, as well as in macrophages, inducing the expression and secretion of pro-inflammatory mediators, such as tumour necrosis factor α (TNFα), and interleukins IL-1β, IL-8 and IL-12 (4,6,7). Asprosin exerts these pro-inflammatory effects via Toll-like receptor 4 (TLR4), Jun N-terminal kinase (JNK) and nuclear factor-kappa B (NFκB) pathways (4,6,7).

Moreover, asprosin exerts orexigenic effects in relation to its role in regulating appetite (4). In terms of its orexigenic function, asprosin is able to cross the blood brain barrier activating agouti-related protein neurons in the hypothalamus. This role is modulated via the same G-protein-cAMP-PKA pathway, although here it is thought to be through a different cell surface receptor, namely the protein tyrosine phosphatase receptor type D (PTPRD) (8,9). Mishra *et al* (8) showed that genetic ablation of this ligand in a mouse model resulted in strong loss of appetite, leanness and lack of response to asprosin's orexigenic effects (8). Notably, emerging studies point towards a role for asprosin in reproduction. For example, asprosin levels are elevated in women with polycystic ovary syndrome (PCOS) (10), gestational diabetes mellitus (GDM) (11), and preeclampsia (12). Asprosin has also been shown to induce markers for ovarian folliculogenesis and steroidogenesis in a mouse model (13). Given the increasing evidence on the pleiotropic effects/roles of asprosin, in the present study, we investigated the potential role of asprosin in relation to placental cells *in vitro*, by assessing its effects on the transcriptome of BeWo and JEG-3 placental cell lines. We have also expanded on our observations by measuring the expression of FBN1/Furin and asprosin's candidate receptors in normal placentas and comparing these to placentas from GDM pregnancies.

## Materials and methods

### Tissue culture

To study placental function *in vitro*, we used two established cell lines, namely the BeWo cell line which secretes hormones and undergoes syncytialisation after treatment with forskolin, and the JEG-3 cell line which does not undergo substantial fusion (14). BeWo cells were cultured using Dulbecco's Modified Eagle's Medium (DMEM) Ham's F12 (Sigma Aldrich D8437) supplemented with 10% foetal Bovine Serum (Sigma Aldrich F6765) and 1% penicillin-streptomycin (Gibco 15140122) at 37°C with 5% CO_2_. JEG-3 cells were cultured using Eagle's minimum essential medium (EBSS) with 2 mM Glutamine, 1% Non-Essential Amino Acids (NEAAs), 1 mM Sodium Pyruvate (NaP), 10% Foetal Bovine Serum (Sigma Aldrich F6765), and 1% penicillin-streptomycin (Gibco 15140122) at 37°C with 5% CO_2_. Both cell lines were treated with 10 nM Recombinant Human Asprosin (BioLegend, 761904) for 4 h. A wound healing assay was also performed, as previously described (15).

### RNA isolation and RNA sequencing

Total RNA was extracted from cell lysates using a Trizol/phenol-chlorophorm based method. Sample purity was assessed using Nano Drop 2000C (Thermo Fisher Scientific, Inc.). Only samples with a ratio of absorbance A260/A280 between 1.8 and 2.1 were used. Triplicate samples were sequenced using the Illumina NextSeq 500/550 Mid Output kit V2.5 (in-house sequencing unit). Data were de-multiplexed and aligned to the human genome. Expression data was analysed using R (v.4.2.3, The R Foundation for Statistical Computinga), with the R studio desktop application (RStudio) and with the use of specific packages; DSeq2 (v1.44), pheatmap and ggplot2. For visualisation, volcano plots were generated using R package ggplot2 (v.3.5.1). Differentially expressed genes (DEGs) were identified for subsequent enrichment analysis.

### Gene expression/gene ontology analysis

The identified DEGs, were then subjected to functional enrichment analysis. Funrich (v3.1.3) (16) was accessed to provide a functional annotation, including biological processes, pathways and molecular functions. Enrichment analysis was also performed using Omics playground (v3.44, BigOmics Analytics) (17) for the function comparison of the genes in asprosin treated versus untreated BeWo and JEG-3 cells.

### Human placental samples

Human placental tissue samples from patients with GDM (n=4; four experimental replicates per sample, i.e., n=16), as well as normal healthy placenta control samples (n=4; three experimental replicates per sample, i.e., n=12) were obtained from the Arden Tissue Bank at the University Hospital Coventry and Warwickshire (UHCW) NHS Trust (ethical approval obtained by the Arden Tissue Bank management committee and by the UHCW ethics committee; NRES 18/SC/0180). Patient consent to participate in the study and use their tissues was obtained, as specified in the Declaration of Helsinki.

Fresh human placental tissue samples were collected on the same day as the delivery/surgery on wet ice in 50 ml Falcon™ tubes full of RNAlater™ stabilization solution (Thermo Fisher Scientific, Inc.). Samples were stored at 4°C until further processing. Paraffin embedded human placental tissue slides were also obtained from the Arden Tissue Bank at UHCW which were collected on the same day as the delivery/surgery and were stored short-term at room temperature until processing.

### Isolation of mRNA, cDNA synthesis and RT-qPCR

Total RNA was extracted from human placental tissue using the Qiagen RNEasy Plus Mini Kit^®^. Sample purity was assessed using Nano Drop 2000C (Thermo Fisher Scientific, Inc.). Only samples with a ratio of A260/A280 between 1.8 and 2.1 were used. cDNA was synthesized using the High-Capacity cDNA Reverse-Transcription Kit (Applied Biosystems™) according to the manufacturer's protocol. The cDNA samples were diluted to 10 ng/µl RNA with nuclease free water and stored at −20°C until further use.

### SYBR Green-based assays

Exploring the expression of a number of genes in both normal healthy and GDM human placental cDNA samples. Primers were obtained from Harvard Primer Bank and RT-qPCR was run using PowerUp SYBR^®^ Green Master Mix. RT-qPCR was performed in triplicate using the primers included in Table I.

### Taqman based-assays

For the purpose of validating the expression of the top genes identified from RNA sequencing, RT-qPCR was performed in triplicate using TaqMan™ Gene Expression Assays (Applied Biosystems) and TaqMan™ Fast Advanced MasterMix (Applied Biosystems). Details on the genes and assay IDs are presented in Table II. All RT-qPCR experiments were carried out on a QuantStudio™ 5 Real-Time PCR System, 96-well (Thermo Fisher Scientific, Inc.). Amplicon load was measured by relative quantification using a ΔΔCq method.

### In silico analysis

ThemiRDB, TargetScan, and ENCORI databases were utilized to identify functional miRNA:*TLR4* interactions (18–20). Following this, the results from these databases were plotted in a Venn diagram using FunRich (21) to identify the more efficacious mRNA target interactions. The database STRING (22) was used to identify the top co-expressed genes with *TLR4*.

### Statistical analysis

Differences identified in experiments were assessed for statistical significance using the unpaired Student's t-test. An assessment for homoscedasticity of data for each data set was made using the F-test. If homoscedasticity was proven, an unpaired Student's t-test was performed to assess significance. If the data were not determined to be homoscedastic, an unpaired Student's t-test with Welch's correction was performed to account for the variance. All statistical tests were performed using GraphPad Prism^®^ software (GraphPad Software, Inc.). P-values <0.05 were considered significant.

## Results

### Effect of asprosin on BeWo and JEG-3 cells

**Figure 1.**
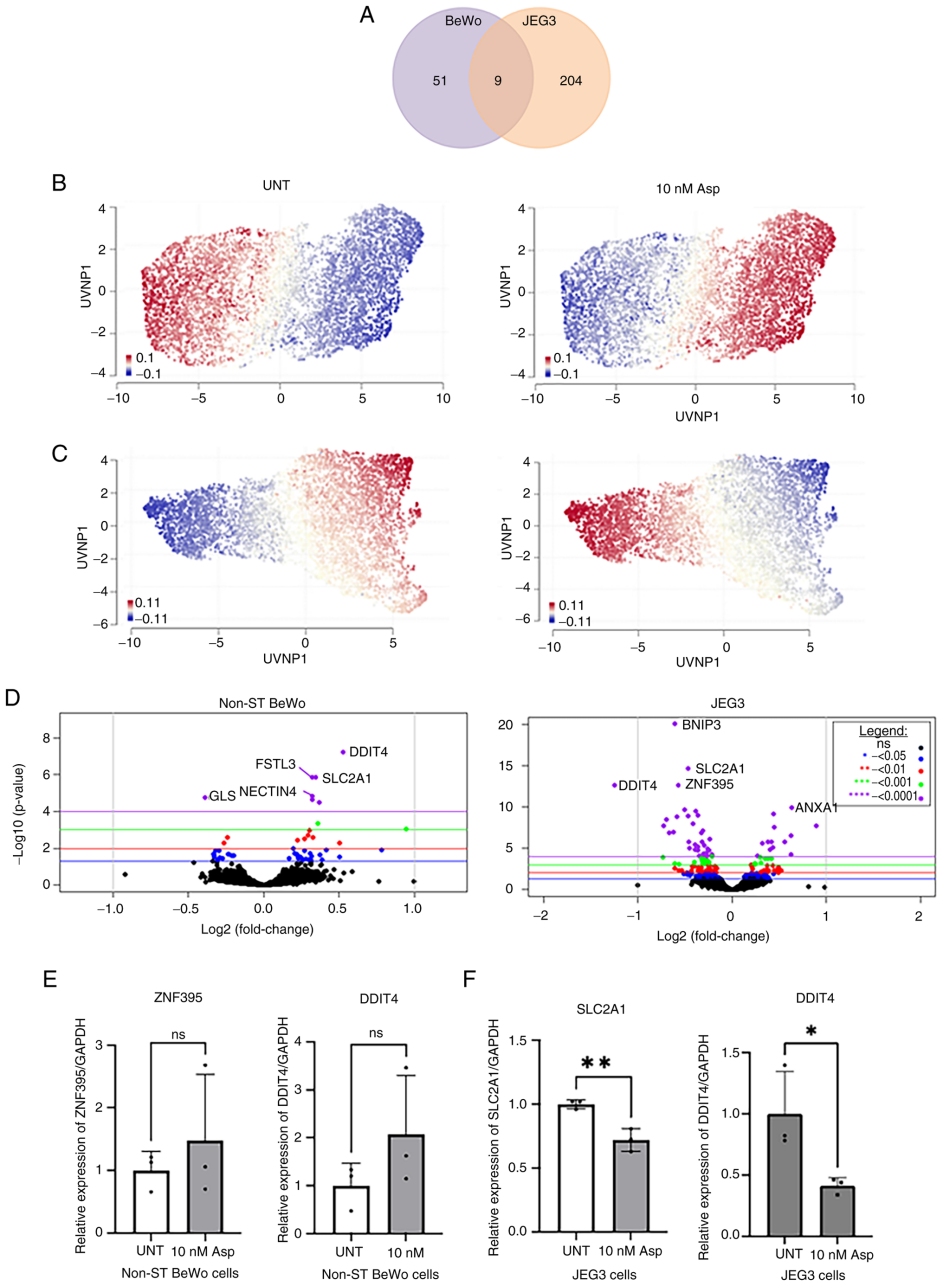
DEGs in BeWo and JEG-3 cells treated with Asp. (A) A Venn diagram displaying the DEGs for Asp-treated BeWo and JEG-3 cells when compared with their respective UNT controls. (B and C) Geneset signature UMAP displaying up- and down-regulated genes in different samples. UMAPs display genes clustered by relative log-expression which were up- (red) or down- (blue) regulated in (B) BeWo and (C) JEG-3 cells, UNT control and 10 nM Asp-treated samples. (D) Volcano plots for BeWo and JEG-3 cells, indicating the most up- and down-regulated DEGs. RNA sequencing validation for (E) BeWo and (F) JEG-3 cells, using reverse transcription-quantitative PCR. *P<0.05, **P<0.001. DEGs, differentially expressed genes; UMAP, uniform manifold approximation and projection; UNT, untreated; Asp, asprosin; ZNF395, Zinc Finger Protein 395; DDIT4, DNA Damage Inducible Transcript 4; SLC2A1, solute carrier family 2 member 1; Non-ST, non-syncytialised; ns, not significant.

To gain a better insight into its role in the human placental transcriptome, BeWo and JEG-3 cells were treated with asprosin (10 nM for 4 h). RNA sequencing revealed that asprosin induced cell specific differential expression for 51 genes (DEGs) in BeWo cells, and 204 in JEG-3 cells, with nine common DEGs in both *in vitro* models (Fig. 1A; Tables SI and SII). A Uniform Manifold Approximation and Projection (UMAP) was constructed to display the up (red) and down-regulated (blue) genes for BeWo and JEG-3 asprosin-treated and control (*i.e*. untreated) samples (Fig. 1B,C). The results show a contrast between the two cell lines in terms of treated and control samples (see volcano plots, Fig. 1D). We have used Taqman probes for validation of RNAseq data, for two up- and down-regulated genes in BeWo (Fig. 1E), and JEG-3 (Fig. 1F) cells, respectively. Despite the lack of significance in BeWo asprosin-treated cells, a trend for upregulation of *ZNF395* and *DDIT4* was noted. In JEG-3 cells, both SLC2A1 and DDIT4 were significantly downregulated following treatment with asprosin (P<0.001 and P<0.05 respectively).

**Figure 2.**
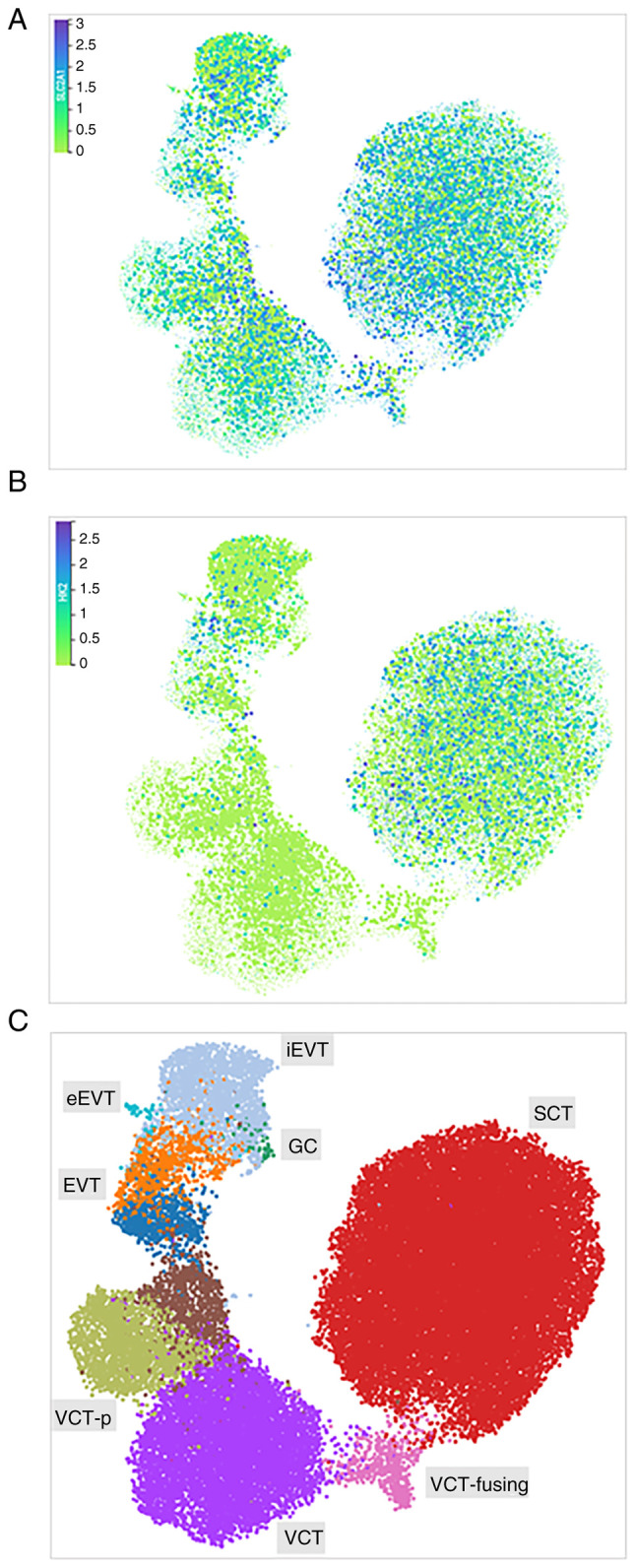
Differential expression of (A) SLCA1 and (B) HK2 using a (C) spatial transcriptomics trophoblast map. SCT, villous syncytiotrophoblast; EVT, extravillous trophoblast cells; eEVT, endovascular trophoblast cells; GC, placenta giant cells; VCT, villous cytotrophoblast; VCT-p, VCT proliferative.

We have used a spatially resolved single-cell multiomic characterization of the maternal-foetal interface (reproductivecellatlas.org) (23) to map the expression of SLCA1 and HK2 in a diverse trophoblast population (Fig. 2). SLCA1 is abundantly expressed in villous syncytiotrophoblasts (SCTs), extravillous trophoblast cells (EVTs), and villous cytotrophoblast cells (VCTs), as well as in placenta giant cells (GCs) (Fig. 2A and C). HK2 expression was primarily confound in SCTs (Fig. 2B and C).

### Heat map of top 50 DEGs identifies four different clusters in both asprosin-treated cell lines

**Figure 3.**
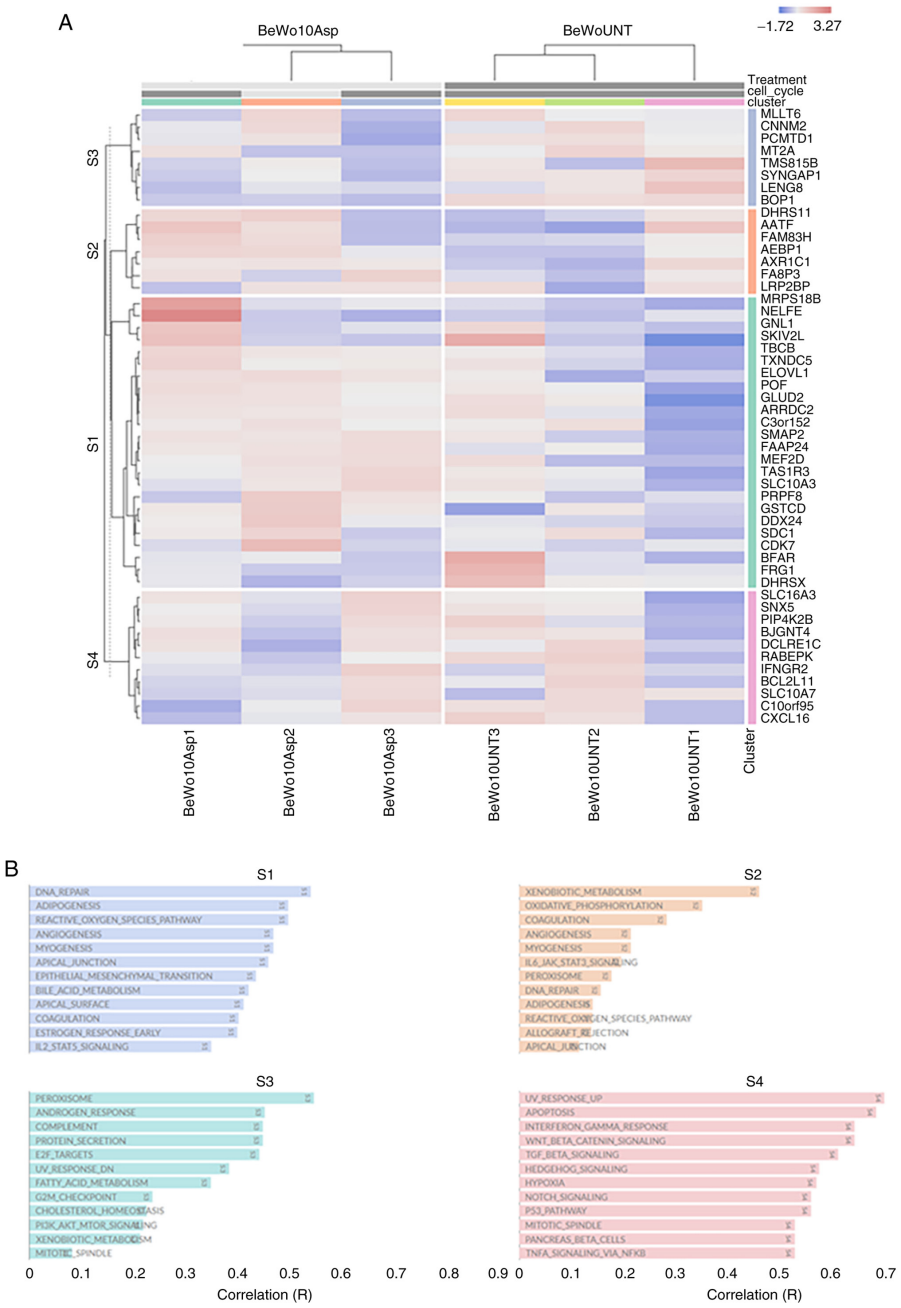
Clustered heatmap and functional annotation. (A) Functional heat map of the top 50 differentially expressed genes identified through highest standard deviation across all samples (BeWo cells asprosin-treated vs. control). The hierarchical clustering was performed at the gene level and using the relative expression scale. In this heatmap, red signifies over-expression and blue under-expression. (B) There are four clusters S1 (blue), S2 (orange), S3 (green) and S4 (red). These were generated using Omics Playground (https://bigomics.ch/omics-playground), which correlates each gene set to over 42 reference databases, such as Kyoto Encyclopaedia of Genes and Genomes and Gene Ontology.

**Figure 4.**
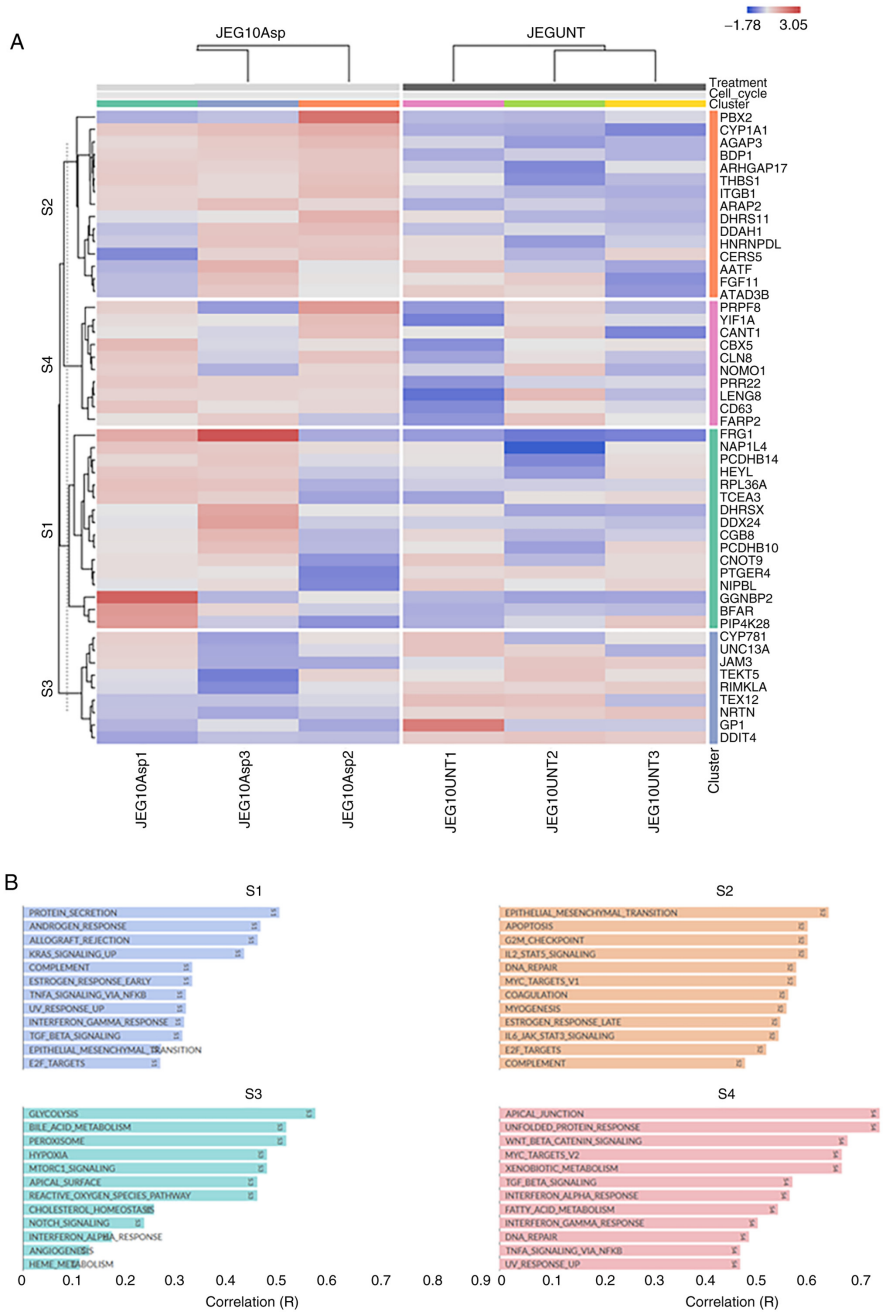
Clustered heatmap and functional annotation. (A) Clustered heatmap and functional annotation of the top 50 differentially expressed genes identified through highest standard deviation across all samples (JEG-3 asprosin-treated vs. control). (B) Subsequently, four distinct clusters S1 (blue), S2 (orange), S3 (green) and S4 (red) were identified using Omics Playground.

Fig. 3A presents the functional heat map of the top 50 DEGs with highest standard deviation across all samples (BeWo-treated *vs*. control). The hierarchical clustering was performed at the gene level and showcased four clusters S1-S4 (Fig. 3B). Many of these functional annotations are related to DNA Repair, angiogenesis, fatty acid metabolism, mTOR/NOTCH/WNT/p53 signalling. Similarly, four distinct clusters were identified in JEG-3 treated samples (Fig. 4A), including changes in protein secretion, glycolysis, TGFβ/TNFα/KRAS/L2 signalling, hypoxia and steroid response (Fig. 4B).

### Gene enrichment analysis

**Figure 5.**
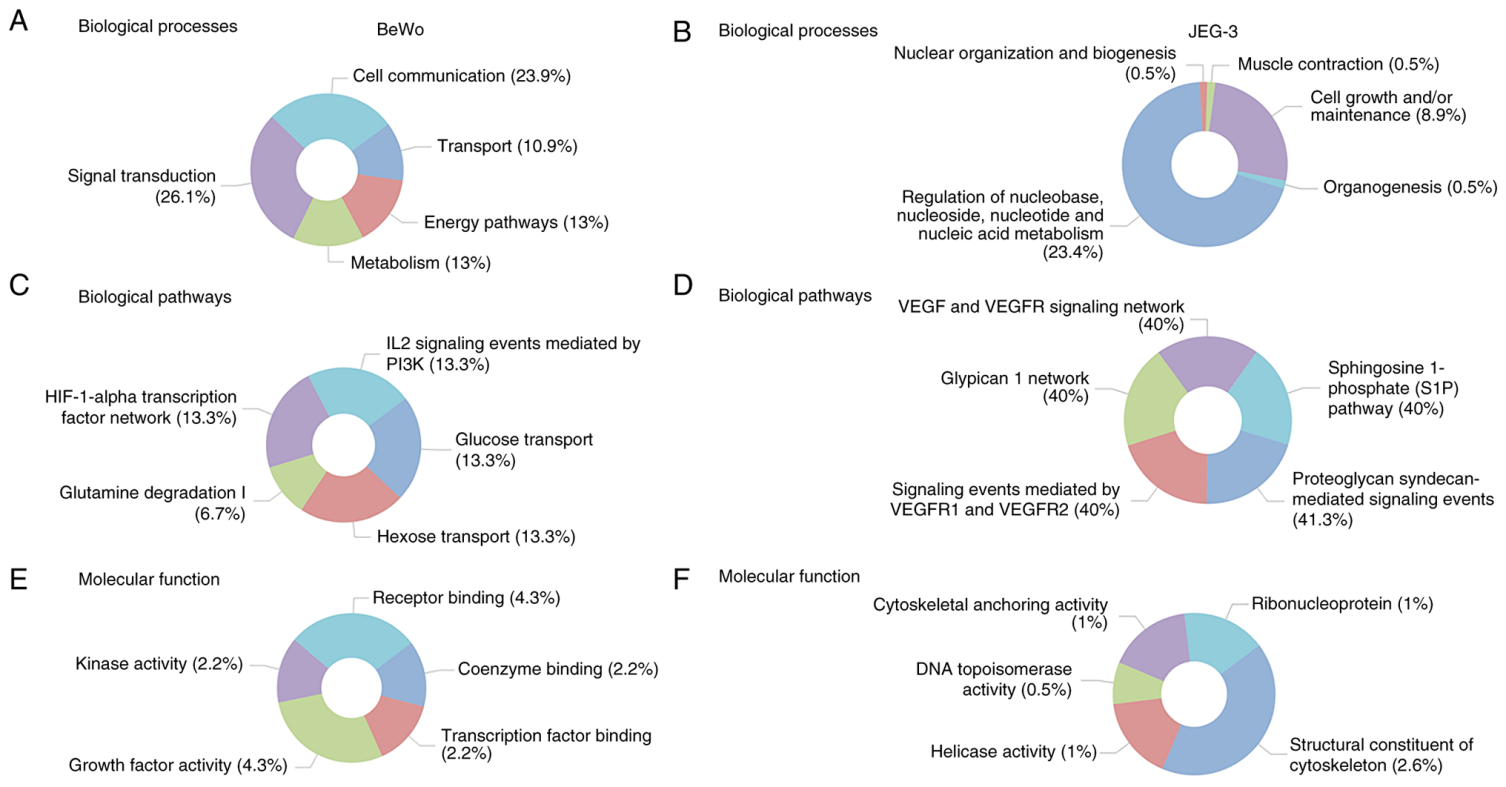
Enriched biological processes, pathways, and molecular functions associated with asprosin treatment. Biological processes of (A) BeWo and (B) JEG-3 cells. Pathways of (C) BeWo and (D) JEG-3 cells. Molecular functions in (E) BeWo and (F) JEG-3 cells.

Funrich was used to determine the top five enriched biological processes (Fig. 5A), biological pathways (Fig. 5B), and molecular functions (Fig. 5C) in BeWo cells treated with asprosin. The main biological processes identified as: signal transduction, cell communication, energy pathways/metabolism, and transport. Glucose/Hexose transport were two of the five biological pathways identified, underpinning the role of asprosin in cell homeostasis. Enriched molecular functions included receptor binding, growth factor and kinase activity.

Similar analyses were performed for the JEG-3 related DEGs, identifying the top five enriched biological processes (Fig. 5D), biological pathways (Fig. 5E), and molecular functions (Fig. 5F). Most enriched biological processes included regulation of nucleic acid metabolism, and cell maintenance, whereas vascular endothelial growth factor receptor (VEGFR) signalling predominated under biological pathways. Contrary to BeWo cells, structural constituent of the cytoskeleton was the most enriched molecular function in JEG-3 cells. All p-values for the top five enriched biological pathways, biological processes, and molecular pathways (depicted in Fig. 5) are presented in Fig. S1.

Furthermore, we have assessed asprosin's role in cell proliferation and migration *in vitro*, using BeWo cells. When cells were treated with asprosin, no apparent differences were observed at 24 or 48 h post asprosin treatment (Fig. S2A). The performed wound healing assay at the same time-points indicated a potential cytostatic effect for asprosin (Fig. S2B and S2C).

### Expression of FBN1, Furin, OR4M1, PTPRD and TLR4 in placentas from normal and GDM pregnancies

**Figure 6.**
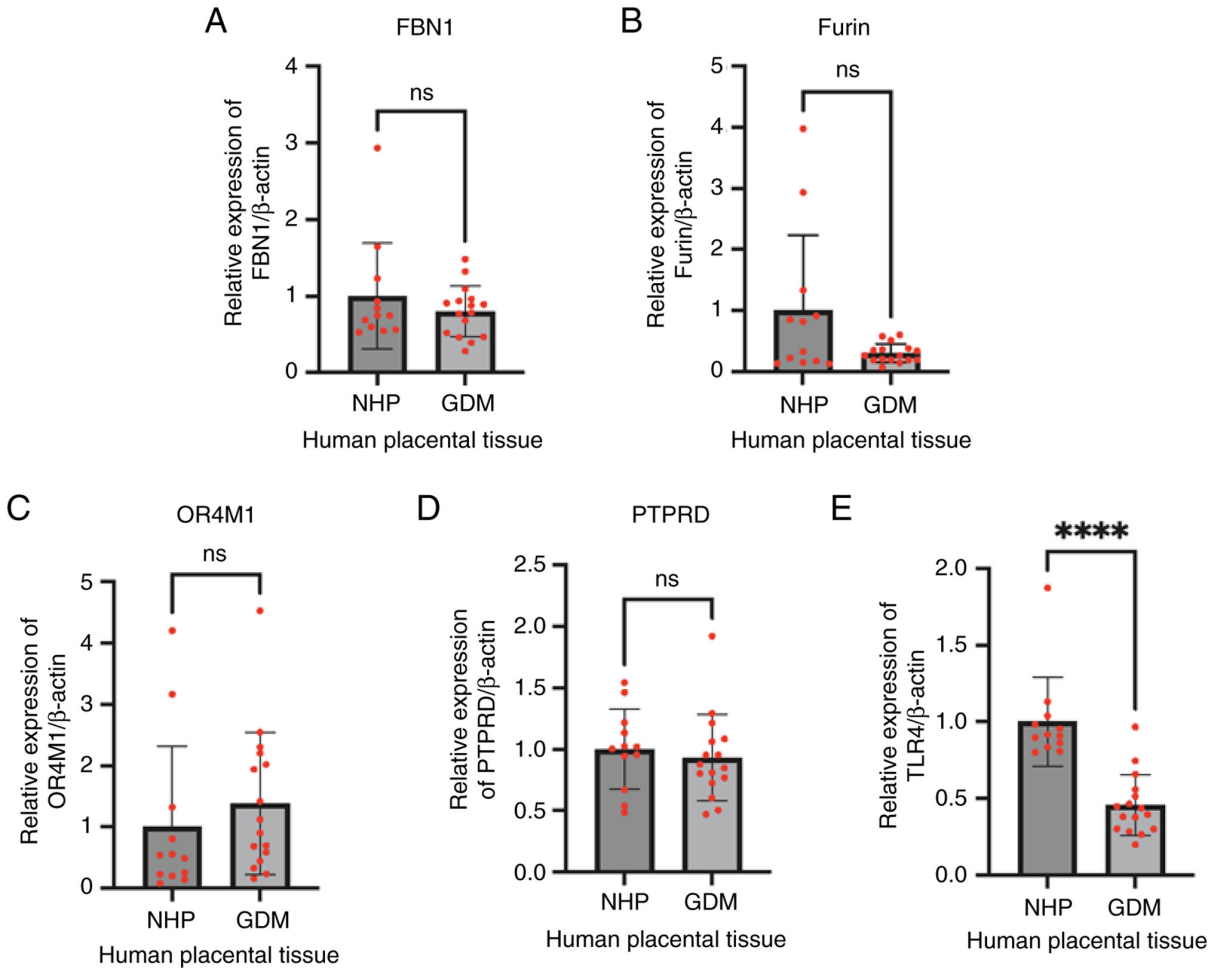
Gene expression of (A) FBN1, (B) Furin, (C) OR4M1, (D) PTPRD and (E) TLR4 in NHP (control) and GDM placenta samples, as assessed by reverse transcription-quantitative PCR. ****P<0.0001. FBN1, fibrillin-1; OR4M1, olfactory receptor 4M1; PTPRD, protein tyrosine phosphatase receptor type D; GDM, gestational diabetes mellitus; NHP, normal human placenta; ns, not significant.

Expression of FBN1, Furin and putative asprosin receptors was assessed in normal and GDM placentas at term. There was no difference in the gene expression of either FBN1 (Fig. 6A) or Furin (Fig. 6B) between these two groups. Similar expression of OR4M1 (Fig. 6C), and PTPRD (Fig. 6D) was also noted between normal and GDM samples, whereas TLR4 expression (Fig. 6E) was significantly downregulated in GDM placentas compared to the controls (P<0.0001).

### Identification of miRNA:mRNA interactions and investigation of TLR4 effects in signalling pathways

Investigation of three predictive miRNA databases was undertaken, namely miRDB, TargetScan, and ENCORI, which identified 95, 1, and 91 interactions, respectively (Fig. S3A). The results showed that there were 10 common miRNAs between ENCORI and miRDB targeting TLR4, and only one common miRNA between miRDB and TargetScan. The 10 common miRNAs are: hsa-miR-448, hsa-miR-642a-5p, hsa-miR-7-5p, hsa-miR-25-3p, hsa-miR-367-3p, hsa-miR-363-3p, hsa-miR-92a-3p, hsa-miR-92b-3p, hsa-miR-32-5p, and hsa-miR-655-3p. The one common miRNA is hsa-miR-140-5p.

Given the most notable changes in receptor expression rest with TLR4, we have performed STRING analysis, and we have identified candidate proteins which have a cross-talk with TLR4. The proteins are: TICAM1, TICAM2, IRAK4, TRAF6, TIRAP, TLR2, LY96, TLR6, HSPD1, and HMGB1. With the exemption of TLR2, all other interactions are experimentally determined (Fig. S3B).

## Discussion

In the present study, we provide evidence of how asprosin can change the placental transcriptome using two well-characterised *in vitro* models. We have also measured the expression of FBN1, the proteolytic enzyme furin, as well as asprosin's putative receptors (OR4M1, PTPRD and TLR4) (9) in healthy (normal pregnancy) and GDM placentas.

Asprosin treatment altered almost 4-fold more genes in JEG-3 cells compared to BeWo cells, indicating their inherent transcriptomic differences, as previously described (24). Of note, nine genes were similarly affected in both these cell lines, namely SLC2A1, ZNF395, DDIT4, HK2, STC2, RGS16, SH3PDXD2B, XYLT1 and CENPF. SLC2A1 encodes a major placental glucose transporter (GLUT1), increases in expression with gestation, and facilitates glucose uptake (25). Similarly, asprosin also affected the expression of Hexokinase 2 (HK2), an enzyme which phosphorylates glucose to glucose-6-phosphate, the first step in most glucose metabolism pathways (26). Both GLUT1 and HK2 appear to be upregulated in patients with GDM (27). Another link between asprosin and glucose is suggested by the differential regulation of stanniocalcin-2 (STC2), a gene which is also upregulated in GDM placentas and inhibits trophoblast invasion under high-glucose conditions (28). DNA Damage Inducible Transcript 4 (DDIT4) has also been affected by asprosin. This is also a crucial signalling pathway in the human placenta which regulates cell proliferation by inhibiting the activity of the mechanistic target of rapamycin (mTOR) (29,30). Notably, it has been suggested that DDIT4 is critical for normal decidualization and possibly involved in the development of preeclampsia (31). For the remaining genes (ZNF395, RGS16, SH3PDXD2B, XYLT1, CENPF) no data are available for specific functions in the human placenta. However, Xylosyltransferase 1 (XYLT1) has been described as an insulin sensitizer (32), whilst depletion of CENPF disrupts GLUT4 trafficking in murine cells (33), and RGS16 induces insulin secretion (34). Collectively, this is the first time that a direct effect of asprosin as a glucose sensor on key components, such as SLC2A1, HK2, and STC2, has been shown at the placental level.

In accordance with their genetic/phenotypic differences as trophoblastic cell lines, BigOmics analytics generated four distinct pathway-related clusters for BeWo and JEG-3 cells based on RNAseq data. Despite some overlap, notable pathways for JEG-3 cells include glycolysis, mTORC1, NOTCH, and KRAS signalling. Common pathways include steroidal responses, cholesterol homeostasis, xenobiotic and fatty acid metabolism, as well as cytokine responses and hypoxia. Glycolysis is an important process for the maintenance of the homeostasis of the maternal-foetal interface, as well as ensuring normal gestation (35). Dysregulation of glycolysis has attracted interest for its role in pregnancy disorders, including miscarriage, GDM and preeclampsia. Indeed, Lu *et al* (36) have shown that NOTCH signalling, glycolysis, and hypoxia were the main enriched area for six preeclampsia-related genes, using the Gene Expression Omnibus public database (36).

We have also explored DEG enrichment for both cell lines in terms of the role of the DEGs in biological processes and pathways, as well as molecular function using FunRich, where a non-overlapping enrichment emerged when the two cell lines were compared. In BeWo cells, the most enriched biological process with mapped genes included GLS, HK2, TMX1, SGMS1, XYLT1 and ILVBL. Corroborating previous data, glucose transport was the most enriched biological pathway (SLC2A1, HK2); whereas the most enriched molecular function was that of growth factor activity, involving GDF15 and PGRN. Interestingly, low expression of growth differentiation factor 15 (GDF15) has been associated with impaired invasion of extravillous trophoblasts and predisposition to pregnancy loss (37), whereas progranulin (PGRN) deficient mice developed abnormal placental angiogenesis (38). In JEG-3 cells, nucleic acid metabolism and VEGF signalling were amongst the most enriched biological processes and pathways. VEGF is a key angiogenic factor that affects not only endothelial cells, but also trophoblasts (39). Impaired glucose tolerance appears to affect the expression of placental VEGFRs (40). Contrary to BeWo cells, ‘structural constituents of the cytoskeleton’ was the most enriched molecular function in JEG-3 cells. For this function, mapped genes included DSP, KRT19, ERRFI1, ACTB and ACTR1A; none of which have any known placental-specific functions assigned.

Of note, HK2 and SLC2A1 expression was noted during embryo morphogenesis (in the period between implantation and gastrulation) (41); particularly in cytotrophoblasts and syncytiotrophoblasts, but not in epiblasts or hypoblasts (see Fig. S4). The presence of these genes as early as nine days post-fertilisation, suggests an important role for embryonic development. Moreover, when cytotrophoblast cells (BeWo cells) were treated with asprosin over 48 h, no apparent changes were noted in cell proliferation, corroborating previous human and animal studies (supplementary data) (42,43). Future studies should use *ex vivo* or *in vivo* models, or even more comprehensive clinical studies to confirm the role(s) and mechanism(s) of asprosin in the overall pregnancy environment.

Following RNA sequencing analysis, we investigated the expression of FBN1, Furin, OR4M1, PTPRD and TLR4 in healthy and GDM placentas. Early studies have shown increased plasma asprosin levels in pregnant women with GDM as early as 18–20 weeks of gestation (44). More recently, Boz *et al* (11) showed that asprosin levels were elevated in pregnant women with normal glucose tolerance or with GDM when compared to healthy non-pregnant controls (11). In the present study, no difference in the expression of FBN1 and Furin was noted between GDM and healthy placentas. This suggests that the source of elevated asprosin in GDM pregnancies is not likely the placenta. Indeed, GDM is associated with elevated maternal body mass index (BMI), so it is possible that increased in adiposity drives higher release of asprosin in circulation. For example, a positive correlation between placental asprosin immunoreactivity and BMI has been shown (45). In terms of the putative asprosin receptors, only TLR4 was significantly downregulated in GDM placentas in our study. Numerous studies have shown that TLR4-mediated signalling plays a pivotal role in immune and inflammatory processes (46). Moreover, a TLR4/NF-κB/PFKFB3 signalling cascade might provide a link between glycometabolism and trophoblastic pyroptosis (47). However, a previous study has reported elevated levels of TLR4 in patients with GDM (48). Thus, more studies are needed to determine the potential differences in the expression of asprosin receptors at both gene and protein level in GDM and other pregnancy complications.

Of note, relating to the identified potential miRNA interactions with TLR4, there is good evidence for involvement of hsa-miR-7-5p, hsa-miR-92a-3p, and hsa-miR-32-5p in GDM/obesity. For example, miRNA 32 and 92a-3p are upregulated in GDM (49,50), whereas hsa-miR-7-5p expression was reduced at 21 days post bariatric surgery (51). STRING motif also revealed interactions implicated in GDM. For example, it has been suggested that the miR-146a-3p/TRAF6 interaction might play a key role in the pathogenesis of GDM (52), whereas HMGB1 expression is increased as a result of tissue damage due to inflammation and oxidative stress related to GDM (53). Following analyses of cord blood samples from diabetic and normal pregnancies, it was shown that maternal diabetes drives a profound inflammatory activation in neonates that involves TLR1/2 or TRL5 (54). It should be noted that there is a further interplay between TLR2 and TLR4, since it has been shown that, under foetal exposure to GDM conditions, TLR4 and TLR2 can activate IL-1β responses in rat offspring spleen cells (55). This corroborates a previous *in vivo* study, where C57BL/6 mice lacking TLR4 (TLR4-knockout, TLR4^−/−^ mice) were partially protected from high-fat diet-induced insulin resistance, suggesting that TLR4 acts as molecular link among pro-inflammatory responses, nutrition, and lipids (56).

To conclude, the present study provides a novel insight into the actions of asprosin in two well-established *in vitro* placental (trophoblast) models, identifying key genes and signalling pathways. Based on the present findings, a common theme that emerged is that of glucose homeostasis, in accordance with the physiologic role of this adipokine. Future work is needed to understand the exact role of asprosin in health and disease (e.g. in GDM) expanding on *in vitro* models (*e.g*., syncytialised BeWo cells), using primary placental cells, as well as trying to recapitulate better the placental microenvironment using 3D cultures.

## Supplementary Material

Supporting Data

Supporting Data

## Figures and Tables

**Table I.** List of primers used for reverse transcription-quantitative PCR in human placenta samples.

Name	Amplicon Size, bp	Strand	Size (bases)	Sequence (5′-3′)
Furin	108	Forward	22	TCGGGGACTATTACCACTTCTG
		Reverse	20	CCAGCCACTGTACTTGAGGC
PTPRDδ	105	Forward	21	CAGGCGGAAGCGTTAATATCA
		Reverse	23	TTGGCATATCATCTTCAGGTGTC
OR4M1	100	Forward	23	TCTGTTAATGTCCTATGCCTTCC
		Reverse	20	AATGTGGGAATAGCAGGTGG
TLR4	94	Forward	23	AGTTGATCTACCAAGCCTTGAGT
		Reverse	23	GCTGGTTGTCCCAAAATCACTTT
FBN1	166	Forward	20	TTTAGCGTCCTACACGAGCC
		Reverse	21	CCATCCAGGGCAACAGTAAGC
Beta Actin	140	Forward	20	CTGGAACGGTGAAGGTGACA
		Reverse	23	AAGGGACTTCCTGTAACAATGCA

**Table II.** List of primers used for RNA sequencing validation.

Gene ID	Gene name	Amplicon Size, bp	Assay ID
DDIT4	DNA damage inducible transcript 4	68	Hs01111686_g1
HK2	Hexokinase II	149	Hs00606086_m1
STC2	Stanniocalcin 2	93	Hs01063215_m1
SLC2A1	Solute carrier family 2 member 1/glucose transporter 1	76	Hs00892681_m1
ZNF395	Zinc finger protein 395	97	Hs00608626_m1
GAPDH	Glyceraldehyde-3-phosphate dehydrogenase	157	Hs02786624_g1

## Data Availability

The data generated in the present study may be requested from the corresponding author.
