# Supplementary material for: Asprosin activates multiple placental pathways in vitro: Evidence for potential involvement in angiogenesis, fatty acid metabolism and the mTOR, NOTCH and WNT signalling pathways
Source: Mol Med Rep. 2025 Sep 4;32(5):309. doi: 10.3892/mmr.2025.13674 (PMC12446899; doi:10.3892/mmr.2025.13674)

Figure S1. Gene Ontology enrichment analysis for differentially expressed genes in (A-C) BeWo and (D-F) JEG-3 cells. (A and D) Biological process, (B and E) Biological pathway and (C and F) Molecular function.

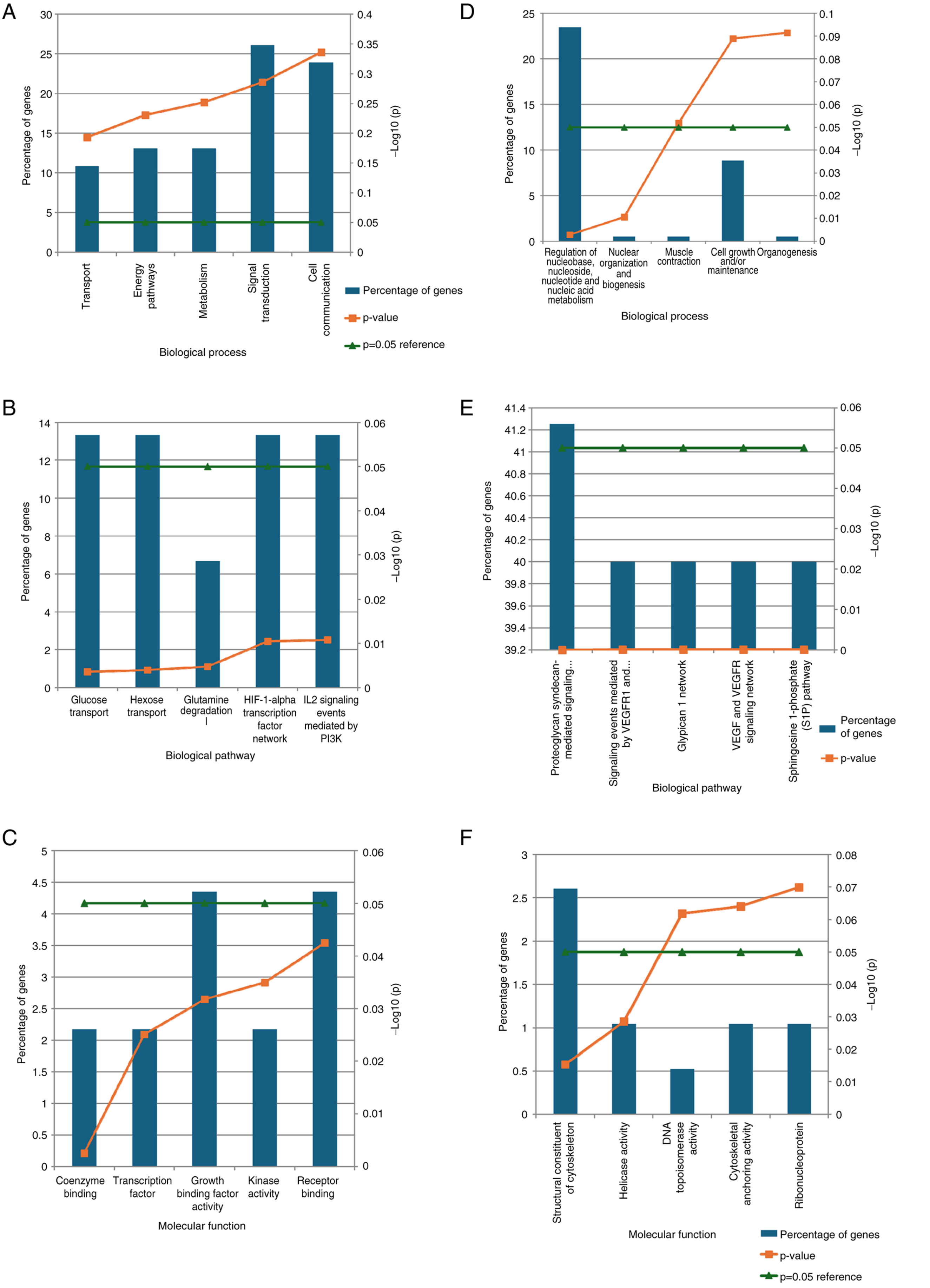

Figure S2. (A) BeWo cell viability following asprosin treatment showed no apparent effect on cell proliferation over time (0-48 h) compared with control, non-treated cells. Wound healing assay of (B) control untreated and (C) BeWo cells treated with asprosin from 24 to 48 h. The gap closure appears to be delayed in the treated cells when compared to control cells (no treatment).

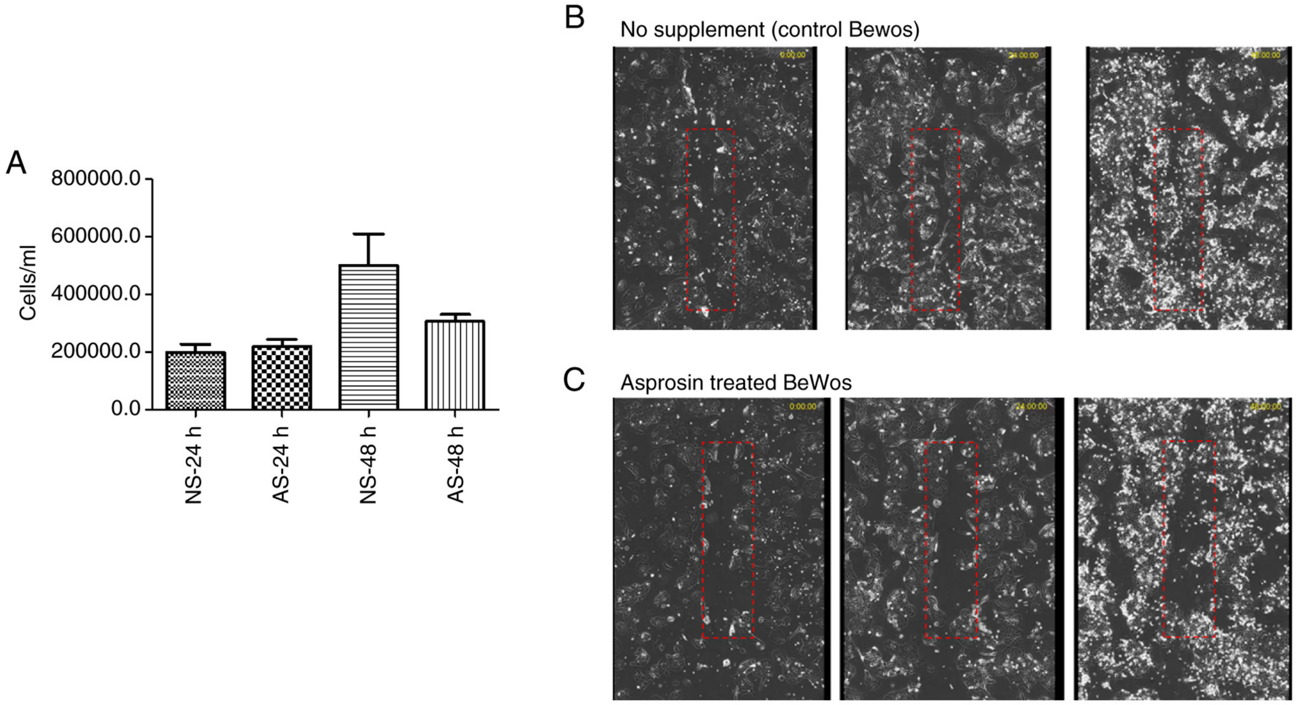

Figure S3. (A) A Venn diagram representing the results from a bioinformatics analysis based on three predictive miRNA-mRNA databases to identify miRNA:TLR4 interactions (miRDB, TargetScan, ENCORI). STRING analysis depicted ten proteins interacting with CIP2A. (B) These are: TICAM1, TICAM2, IRAK4, TRAF6, TIRAP, TLR2, LY96, TLR6, HSPD1 and HMGB.

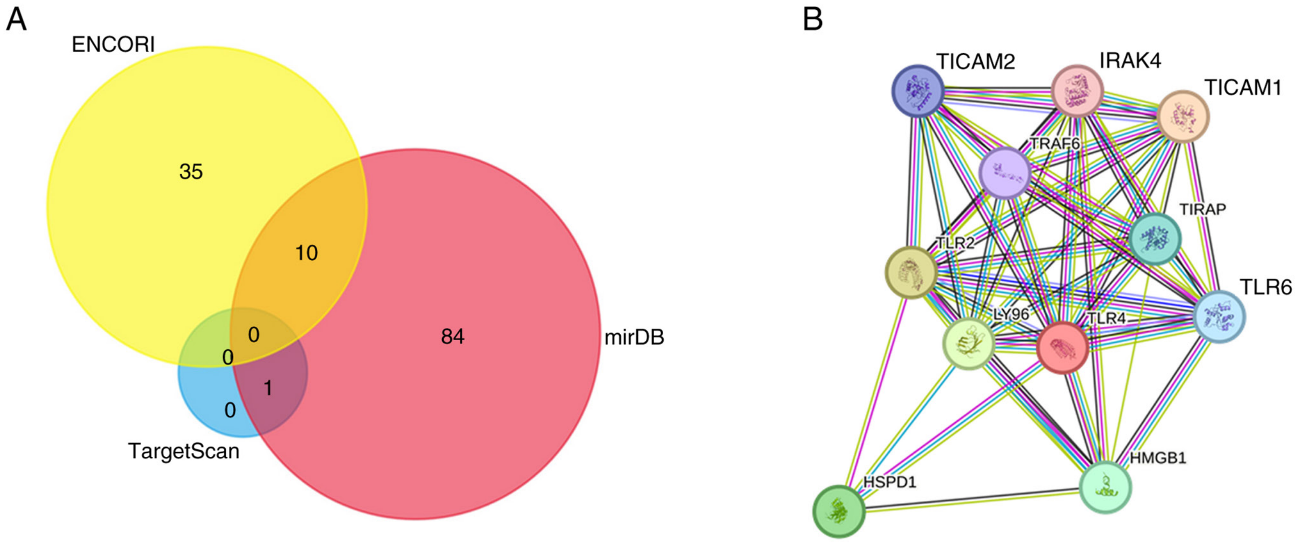

Figure S4. Mapping of HK2 and SLC2A1 during embryonic development. (A) The four major clusters coloured by lineage via UMAP: Epiblast (GREEN-1; 108 cells at 9 d.p.f.; 58 cells at 11 d.p.f.; total=166), hypoblast (CYAN-2; 82 cells at 9 d.p.f.; 54 cells at 11 d.p.f.; total=136), cytotrophoblast (RED-3; 1,956 cells at 9 d.p.f; 226 cells at 11 d.p.f; total=2,182), syncytiotrophoblast (PURPLE-4; 1,058 cells 9 d.p.f.; 1,278 cells at 11 d.p.f.; total=2,336). Expression of (B) HK2 and (C) SLC2A1 in these four clusters. HK2, hexokinase 2; SLC2A1, solute carrier family 2 member 1; d.pf., days post-fertilisation.

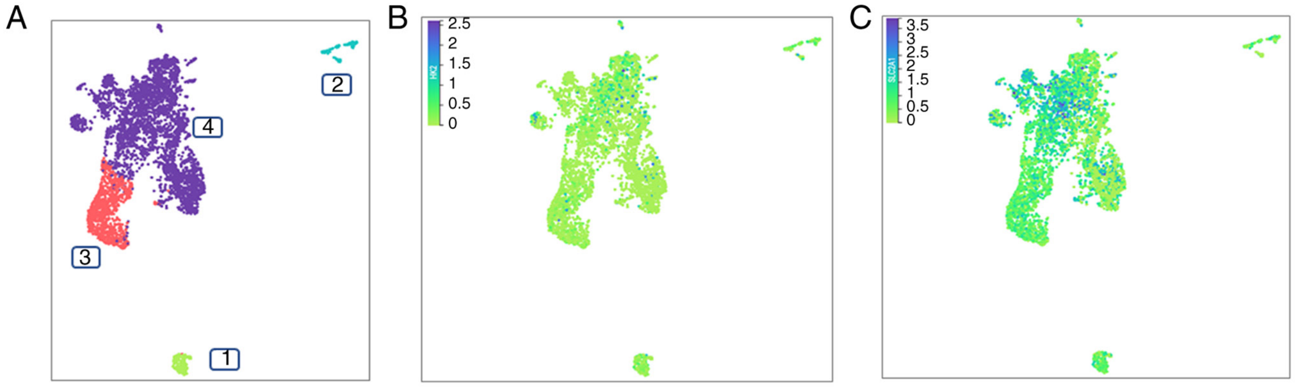

Supplement: Supporting Data [file Supplementary_Data1.pdf]
