# Supplementary material for: Asprosin activates multiple placental pathways in vitro: Evidence for potential involvement in angiogenesis, fatty acid metabolism and the mTOR, NOTCH and WNT signalling pathways
Source: Mol Med Rep. 2025 Sep 4;32(5):309. doi: 10.3892/mmr.2025.13674 (PMC12446899; doi:10.3892/mmr.2025.13674)
Supplement: Supporting Data [file Supplementary_Data2.pdf]

Table S1. List of differentially expressed genes of BeWo asprosin-treated cells.

NM Number	Gene
NM_019058	DDIT4
NM_005860	FSTL3
NM_006516	SLC2A1
NM_030916	NECTIN4
NM_001256310	GLS
NM_021939	FKBP10
NM_018660	ZNF395
NM_002928	RGS16
NR_027260	RN7SL2
NM_017789	SEMA4C
NM_138383	MTSS2
NM_003400	XPO1
NM_130807	MOB3A
NM_003714	STC2
NM_000189	HK2
NM_178493	NOTUM
NM_033505	SELENOI
NM_017510	TMED9
NM_020650	RCN3
NR_002715	RN7SL1
NM_003566	EEA1
NM_016545	IER5
NM_017801	CMTM6
NM_004864	GDF15
NM_031476	CRISPLD2
NM_012288	TRAM2
NM_016343	CENPF
NM_016531	KLF3
NM_030755	TMX1
NM_153690	FAM43A
NM_005567	LGALS3BP
NM_001071775	MZT1
NM_017892	PRPF40A
NM_052905	FMNL2
NM_001017995	SH3PXD2B
NM_021623	PLEKHA2
NM_001144952	SDK2
NM_147156	SGMS1
NM_006854	KDELR2
NM_002087	GRN
NM_020726	NLN

NM_021149	COTL1
NM_024329	EFHD2
NM_178815	ARL5B
NM_005895	GOLGA3
NM_003394	WNT10B
NM_006423	RABAC1
NM_015111	N4BP3
NM_022166	XYLT1
NM_006844	ILVBL
NM_018353	MIS18BP1

Table II. List of differentially expressed genes of JEG-3 asprosin-treated cells.

NM Number:	Gene:
NM_004052	BNIP3
NM_006516	SLC2A1
NM_018660	ZNF395
NM_019058	DDIT4
NM_000700	ANXA1
NM_004331	BNIP3L
NM_198182	GRHL1
NM_005911	MAT2A
NM_000189	HK2
NM_145257	CCSAP
NM_017644	KLHL24
NM_002166	ID2
NM_004415	DSP
NM_001795	CDH5
NM_022051	EGLN1
NM_005415	SLC20A1
NM_001620	AHNAK
NM_153252	BRWD3
NM_002332	LRP1
NM_004354	CCNG2
NM_017922	PRPF39
NM_006931	SLC2A3
NM_002276	KRT19
NM_005558	LAD1
NM_080725	SRXN1
NM_015021	ZNF292
NM_016639	TNFRSF12A
NM_001012642	GRAMD2A
NM_030625	TET1
NM_003714	STC2
NM_006763	BTG2
NM_002467	MYC
NR_024278	LOC646762
NM_006310	NPEPPS
NM_006328	RBM14
NM_006909	RASGRF2

NM_014877	HELZ
NM_005559	LAMA1
NM_014819	PJA2
NM_001376	DYNC1H1
NM_002521	NPPB
NM_001961	EEF2
NM_031407	HUWE1
NM_000876	IGF2R
NM_005686	SOX13
NM_014423	AFF4
NM_002928	RGS16
NM_018948	ERRFI1
NM_006813	PNRC1
NM_024769	CLMP
NM_004669	CLIC3
NM_020338	ZMIZ1
NM_001287491	TET3
NM_015169	RRS1
NM_139265	EHD4
NM_001999	FBN2
NM_001605	AARS1
NM_015057	MYCBP2
NM_016604	KDM3B
NM_020143	PNO1
NR_026846	ANAPC1P2
NM_021239	RBM25
NM_001013690	FIGNL2
NM_001017995	SH3PXD2B
NM_017798	YTHDF1
NM_001080477	TENM3
NM_012098	ANGPTL2
NM_012341	GTPBP4
NM_015315	LARP1
NM_001283	AP1S1
NM_004525	LRP2
NM_031918	KLF16
NM_025090	

NM_014865	NCAPD2
NM_015131	WDR43
NM_002293	LAMC1
NM_000435	NOTCH3
NM_004491	ARHGAP35
NM_014681	DHX34
NM_170606	KMT2C
NM_001010854	TTC7B
NM_020979	SH2B2
NM_014584	ERO1A
NM_000291	PGK1
NM_057175	NAA15
NM_004907	IER2
NM_014616	ATP11B
NM_002428	MMP15
NM_152230	IPMK
NM_019593	GPCPD1
NM_057749	CCNE2
NM_020702	MYORG
NM_015123	FRMD4B
NM_018697	LANCL2
NM_007124	UTRN
NM_014331	SLC7A11
NM_020733	HEG1
NM_006305	ANP32A
NM_020343	RALGAPA2
NM_012382	TTC33
NM_002111	HTT
NM_002773	PRSS8
NM_003966	SEMA5A
NM_032860	LTV1
NM_014935	PLEKHA6
NM_016530	RAB8B
NM_006029	PNMA1
NM_001067	TOP2A
NM_016133	INSIG2
NM_177538	CYP20A1

NM_004289	NFE2L3
NM_002372	MAN2A1
NM_001271	CHD2
NM_004237	TRIP13
NM_006828	ASCC3
NM_006343	MERTK
NM_001256071	RNF213
NM_021203	SRPRB
NM_001308147	PLEKHG3
NM_022166	XYLT1
NM_033274	ADAM19
NM_006513	SARS1
NM_002824	PTMS
NM_015074	KIF1B
NM_016183	MRTO4
NM_005120	MED12
NM_020357	PCNP
NM_001042414	PSPC1
NM_001080450	BEND3
NM_005990	STK10
NM_015033	FNBP1
NM_017934	PHIP
NM_020774	MIB1
NM_024602	HECTD3
NM_015562	UBXN7
NM_002755	MAP2K1
NM_017643	MBTD1
NM_080677	DYNLL2
NM_004878	PTGES
NM_013319	UBIAD1
NM_022366	TFB2M
NM_032221	CHD6
NM_007225	NXPH3
NR_037427	MIR3654
NM_016243	CYB5R1
NM_001551	IGBP1
NM_018011	ARGLU1

NM_015306	USP24
NM_014597	DNTTIP2
NM_022917	NOL6
NM_002871	RABIF
NM_005220	DLX3
NM_015457	ZDHHC5
NM_145307	RTKN2
NM_001031711	ERGIC1
NM_001101	ACTB
NM_015474	SAMHD1
NM_005044	PRKX
NM_005736	ACTR1A
NM_138809	CMBL
NM_014611	MDN1
NM_015104	ATG2A
NM_147128	ZNRF2
NM_001017964	YDJC
NM_001690	ATP6V1A
NM_001861	COX4I1
NM_053056	CCND1
NM_024334	TMEM43
NM_005066	SFPQ
NM_005642	TAF7
NM_012479	YWHAG
NM_017882	CLN6
NM_015056	RRP1B
NM_002687	PNN
NM_002999	SDC4
NM_013285	GNL2
NM_018351	FGD6
NM_002077	GOLGA1
NM_006784	WDR3
NM_014851	KLHL21
NM_001404	EEF1G
NM_003276	TMPO
NM_014390	SND1
NM_003489	NRIP1

NM_020947	MEAK7
NM_001303095	ISG20L2
NM_025077	TOE1
NM_032329	ING5
NM_203453	PLPP6
NM_175058	PLEKHA7
NM_016252	BIRC6
NM_003370	VASP
NM_006526	ZNF217
NM_005184	CALM3
NM_002205	ITGA5
NM_002079	GOT1
NM_006773	DDX18
NM_021194	SLC30A1
NM_005253	FOSL2
NM_003222	TFAP2C
NM_020459	PAIP2B
NM_016343	CENPF
NM_152307	TRMT61A
NM_022491	SUDS3
